# Advanced Understanding of Monogenic Inflammatory Bowel Disease

**DOI:** 10.3389/fped.2020.618918

**Published:** 2021-01-22

**Authors:** Ryusuke Nambu, Aleixo M. Muise

**Affiliations:** ^1^Cell Biology Program, Research Institute, The Hospital for Sick Children, Toronto, ON, Canada; ^2^SickKids Inflammatory Bowel Disease Center, The Hospital for Sick Children, Toronto, ON, Canada; ^3^Department of Gastroenterology and Hepatology, Saitama Children's Medical Center, Saitama, Japan; ^4^Department of Pediatrics, Institute of Medical Science and Biochemistry, The Hospital for Sick Children, University of Toronto, Toronto, ON, Canada

**Keywords:** monogenic IBD, VEOIBD, inflammatory bowel disease, whole exome sequences, pediatric, genetics

## Abstract

Inflammatory bowel disease (IBD) is a group of chronic disorders that cause relapsing inflammation in the gastrointestinal tract and comprise three major subgroups of Crohn's disease (CD), ulcerative colitis (UC), and IBD-unclassified (IBDU). Recent advances in genomic technologies have furthered our understanding of IBD pathogenesis. It includes differentiation rare monogenic disorders exhibiting IBD and IBD-like inflammation (monogenic IBD) from patients with the common polygenic form of IBD. Several novel genes responsible for monogenic IBD have been elucidated, and the number of reports has increased due to advancements in molecular functional analysis. Identification of these pathogenic genetic mutations has helped in elucidating the details of the immune response associated with gastrointestinal inflammation and in providing individualized treatments for patients with severe IBD that is often unresponsive to conventional therapy. The majority of monogenic IBD studies have focused on young children diagnosed <6 years of age (very early-onset IBD); however, a recent study revealed high prevalence of monogenic IBD in older children aged >6 years of age as well. Meanwhile, although patients with monogenic IBD generally show co-morbidities and/or extraintestinal manifestation at the time of diagnosis, cases of IBD developing as the initial symptom with unremarkable prodromal symptoms have been reported. It is crucial that the physicians properly match genetic analytical data with clinical diagnosis and/or differential diagnosis. In this review, we summarize the essential clues that may physicians make a correct diagnosis of monogenic disease, including classification, prevalence and clinical phenotype based on available literatures.

## Introduction

Inflammatory bowel disease (IBD) is a group of chronic disorders that cause relapsing inflammation in the gastrointestinal tract. They traditionally comprise three major subgroups: Crohn's disease (CD), ulcerative colitis (UC), and IBD-unclassified (IBDU) (1). Recent evidences indicates that IBD is more heterogeneous than has traditionally been recognized using these three major categories (2, 3). One of most important pieces of evidence is the existence of monogenic IBD, which is caused by single gene defects. Translational research involving monogenic IBD has proceeded rapidly over the past few years, and there have been several reports of novel single gene mutations causing monogenic IBD. Based on the literature, we summarize issues of fundamental importance to physicians, including understanding of the genetic basis of these conditions, prevalence and the clinical phenotypes in this review.

## Genetics of IBD

### Monogenic IBD With Mendelian Pattern

Recent advances in genomic technologies, such as next-generation sequencing, have made it possible to diagnose severe refractory IBD and IBD-like disease as rare monogenic disorders. IBD and IBD-like diseases caused by monogenic disorders transmitted according to Mendelian inheritance patterns have been described as “monogenic” IBD, in contrast to “polygenic,” or classical IBD. The majority of monogenic IBD cases occur in children diagnosed under 6 years of age. Glocker et al. first identified interleukin IL-10 receptor deficiencies in young infants with severe IBD and perianal disease (4). Since then, several novel genes responsible for monogenic IBD have been identified. The current number of monogenic IBD disorders continues to grow with genes classified into six categories according to the biologic mechanism including: defects in the epithelial barrier; T- and B-cell defects; hyperinflammatory and autoinflammatory disorders; phagocytic defects; immunoregulation, including IL-10 signaling defects; and other (5).

**Figure 1:**
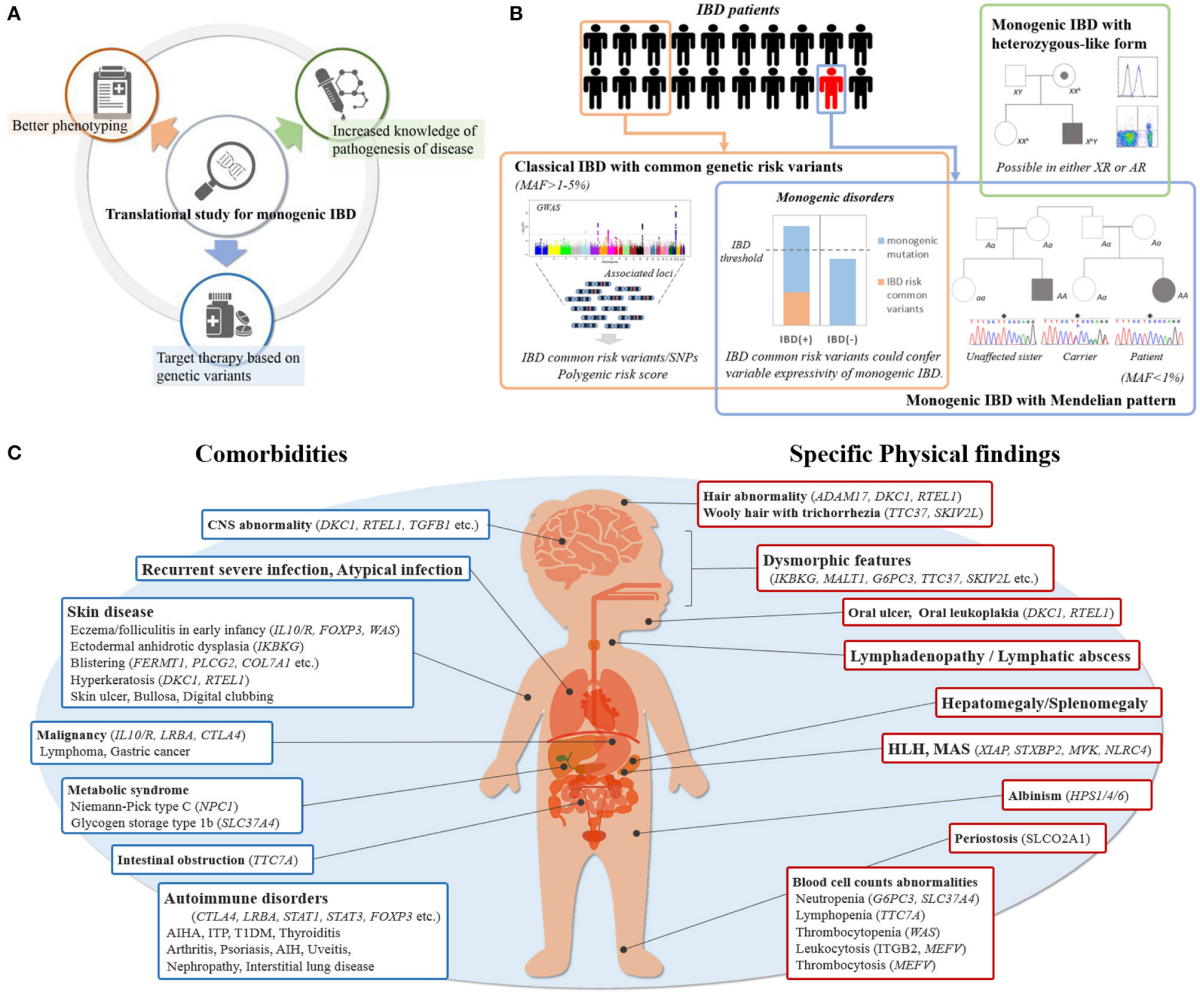
**(A)** Contribution by translational study for monogenic IBD. **(B)** Etiology of pediatric IBD with rare variants. Common genetic IBD risk variants are seen in some of patients, which show classical IBD. Pathogenic rare variants are seen in only a few of patients with IBD, which also has monogenic disorders in accordance with Mendelian pattern. Common genetic IBD risk variants may modulate the effects of pathogenic rare variants. The patients with only single-allele mutations causing monogenic disorders are rarely seen especially in using WES despite generally being X-linked or autosomal recessive. **(C)** Key indicators of monogenic IBD in clinical practice. Showing physical findings and comorbidities of which physicians should be aware at the initial physical examination and during follow-up.

The recent advances in translational research into monogenic IBD has resulted in three major contributions to IBD pathogenesis and care including: (1) increased knowledge of pathogenesis of disease, (2) better phenotyping, (3) targeted therapy based on genetic variants (Figure 1A). First, it has revealed the role of novel protein-coding genes in the intestinal tract, their function in intestinal homeostasis, and several immunological pathways involved in the control of intestinal inflammation. Second, it has elucidated more detailed descriptions of the clinical phenotype and clinical course of monogenic IBD. Third, it has made it possible to provide appropriate treatment for those children with monogenic IBD refractory to standard treatment, and to avoid the necessity for surgery (6, 7). For example, hematopoietic stem cell transplantation (HSCT) for patients with an X-linked inhibitor of apoptosis protein (XIAP) deficiency, FOXP3 deficiency, and chronic granulomatous diseases (CGDs) can improve the prognoses of their IBD (8–10). HSCT has been shown to be ineffective for epithelial barrier dysfunctions, such as TTC7A deficiency and nuclear factor-kappa B essential modulator (NEMO) deficiency (11, 12). HSCT can correct immune defects, but it cannot change the expression of these protein on the intestinal epithelium which is outside the hematopoietic compartment. On the other hand, some monogenic disorders can be treated with medications not commonly used for IBD patients. For example, IL-1 receptor antagonist has been shown to have benefits for patients with mevalonate kinase deficiency (13). CTLA4-Ig immunoglobulin fusion protein (abatacept) is effective for patients with CTLA4 deficiency and lipopolysaccharide-responsive and beige-like anchor protein (LRBA) deficiency (14, 15). This is because LRBA deficiency results in very low CTLA4 expression, which explains the clinical overlap between LRBA- and CTLA4- deficiency. Granulocyte-colony stimulating factor for IBD with glycogen storage disease type 1b (GSD-1b) caused by a mutation in SLC37A4 and colchicine for Familial Mediterranean fever caused by a mutation in MEFV, are commonly used (16, 17).

Recent developments in scientific technology have led to the production of new drugs affecting the immunological pathways of intractable monogenic IBD. Using a phenotypic high-throughput drug screen, Jardine et al. showed that leflunomide could be used in TTC7A-deficient patient-derived colonoids. Clinical trial are currently underway (18).

### Common Genetic Risk Variants for IBD

The impact of the genetics for classical IBD (polygenic IBD) is also significant and different from that of monogenic IBD. Genome-wide association studies (GWAS) have identified about 240 disease loci linked to classical adult-onset IBD and have substantially expanded our understanding of the genetic architecture and causative mechanisms of IBD (Figure 1B) (19, 20). Similarly, common IBD variants associated with pediatric-onset IBD have been investigated; most of these variants are shared with adult-onset IBD (21, 22). On the other hand, the common variants with risk in children with very-early onset (VEO)-IBD (those diagnosed at younger than 6 years of age) have not been cleared in detail.

While these GWAS studies have identified important pathways associated with IBD including autophagy, a clear understanding of the interactions of IBD risk loci and causal genes as most of these GWAS loci are in introns or intergenic regions. Additionally, the majority of common genetic risk polymorphisms detected by GWAS have small effect (odds ratio <1.5). Statistical analysis indicated that these variants could explain only 13% and 8% of the variance in disease heritability for CD and UC, respectively (23). In the future, meta-analysis research combined with transcriptome analysis, epigenetics, and GWAS data are expected to increase our understanding of these regions.

### Expressivity of Monogenic IBD

Variable expressivity is defined as the degree of variation of the clinical phenotype in individuals with monogenic disorders. Expressivity is different from penetrance, a term referring to whether the patient has the clinical phenotype associated with a genetic modification (24). The expressivity of the IBD phenotype in monogenic IBD is often incomplete in both autosomal dominant and autosomal recessive disorders. For example, around 20–30% of patients with XIAP deficiency or with GSD-1b caused by SLC374A4 demonstrate an IBD-like phenotype (16, 25, 26) and 4–9% with Wiskott Aldrich Syndrome develop IBD (27, 28).

Incomplete expressivity is assumed to result from the effects of unlinked modifier genes, epigenetic changes, or environmental factors (24). Recently, common IBD risk variants have been reported to play a significant role in this expressivity (Figure 1B). Tronstad et al. reported GWAS meta-analysis in 22 patients with familial GUCY2C diarrhea syndrome (FGDS), caused by a mutation in GUCY2C. Of 22 FGDS patients, eight showed CD-like phenotype. Seven of the eight patients had either homozygous or heterozygous NOD2 variants, known to be IBD risk, compared with only two of 14 FGDS patients without IBD (29). Huang et al. showed CGD patients with IBD had on average a significantly greater number of variants associated with IBD risk than CGD patients without IBD (30). Thus, common genetic risk polymorphisms for IBD may modify the expressivity of monogenic IBD.

### Monogenic IBD With Heterozygous-Like Form Inheritance

Monogenic IBD usually exhibits a Mendelian inheritance pattern, but some monogenic IBD disorders that are inherited as X-linked or autosomal recessive (Figure 1B) may have exceptions. For example, X-linked CGD may be observed in females CYBB female carrier as a result of X chromosome inactivation resulting in manifestation of the primary immunodeficiency (31). Also, Aguilar et al. reported two female cases of IBD patients with a heterozygous XIAP mutation (32).

Autosomal recessive forms of inheritance are different from X-linked inheritance. When using whole exome sequences (WES), we rarely find only single-allele mutations causing monogenic disorders, although the phenotypes may be compatible with monogenic disorders. There are potential explanations for this phenomenon: (1) the existence of copy-number variation or low frequency gene mosaicism, which may be missed by WES, (2) a second-hit allele may be located in an intron or a non-coding region, altering splice site recognition; or (3) unknown modifier genes may affect penetrance. Regardless of the explanation, an approach to determine whether a variant is disease causing, such as additional sequencing and functional analysis are necessary (Table 1A). For example, Wright et al. reported the case of a 4 years-old boy with IBD caused by a rare pathogenic heterozygote variant in the NCF4 gene (34). This variant was inherited from his healthy father. NCF4, which encodes p40-phox, and normally has an autosomal recessive form of inheritance, is classified as a genetic subgroup of CGD. NADPH oxidase activity in this patient's neutrophils was decreased, as were monocyte and phagocyte killing of bacteria. This patient underwent HSCT and improved without any conventional therapy for IBD. These results suggested that this heterozygote variant led to a defect in neutrophil function and indicated that any other factors are changing the penetrance or expressivity of this variant in this patient, such as may be explained by one of the three hypotheses listed above. Wright et al. suggested that this might be due to the presence of unlinked modifier genes in this report.

**Table 1A:** Functional analysis.

**Functional screening considered**	
• Immunoglobulins (IgG, IgM, IgA, IgD, IgE) • Lymphocyte subsets by flow • Antibody to vaccines • TRECs • DHR-123 • Cytokine assay (Serum cytokine level during flare)	
**Targeted functional analysis (*****recommended*****)**	
***Gene***	***Functional analysis***
*IL10RA*	IL10-induced STAT3 phosphorylation by flow cytometry or immunoblotting
*IL10RB*	
*NCF1*	Neutrophil oxidative burst study, DHR-123 test
*NCF2*	
*CYBA*	
*CYBB*	
NCF4^*^	Neutrophil oxidative burst study
*CYBC1*	
*TTC7A*	Immunohistochemistry-TTC7A, apoptosis
*WAS*	WASP expression by flow cytometry
*XIAP*	XIAP expression by flow cytometry TNF, IL-8, and MCP-1 expression in response to MDP stimulation
*SLCO2A1*	Immunohistochemistry-SLCO2A1
*NPC1*	Filipin staining of cultured skin fibroblasts
*SLC37A4*	G6Pase enzyme activity in Liver tissue (non-frozen)
*MVK*	Increased urine mevalonic acid when fever
*TNFAIP3*	A20 expression by immunoblotting RT-PCR using total RNA
*CTLA4*	CTLA-4 expression within stimulated Treg cells by flow cytometry
*LRBA*	LRBA expression in response to PHA stimulation by flow cytometry
*FOXP3*	FOXP3 expression by flow cytometry
*STAT1*(GOF)	CD25 expression by flow cytometry
*STAT3*(GOF)	STAT3 reporter luciferase assay under basal or stimulated condition (IL-6/growth hormone) in cell lines SOCS3 expression levels under basal or stimulated condition (IL-21) in EBV-transformed patient cell lines

* *, The production of ROS in phagocyte is normal, and need to examine the bacterial killing activity (33)*.

*TREC, T-cell receptor excision circles; DHR-123, dihydrorhodamine 123; WASP, Wiskott-Aldrich Syndrome protein; IL-8, Interleukin-8; MCP-1, monocyte chemoattractant protein; MDP, muramyl dipeptide; RT-PCR, real-time reverse transcription polymerase chain reaction, Treg, regulatory T cell; PHA, Phytohaemagglutinin; SOCS3, suppressor of cytokine signaling 3; EBV, Epstein-Barr virus; ROS, reactive oxygen species*.

Even if only a single-allele mutation causing monogenic disorder is found, the clinicians should consider additional functional test if the patient's phenotype is consistent with that disorder.

## Prevalence of Monogenic IBD in Pediatric IBD Patients

### IBD Onset Before 6 Years of Age

In recent years, several cohort studies have been conducted, using WES and targeted genome panel sequence (TGPS) to determine the prevalence of monogenic IBD in children (Table 1B) (35–41). Among these previous studies, the prevalence of monogenic IBD in children with IBD has varied greatly. This may be due to differences in locality, differences in referral populations, and differences in the feature of the institution. Besides, the discrepancies in allele distribution among ethnic groups, and the cultural and social backgrounds of the regions, which could be influenced by factors such as consanguineous marriage might have an impact on the difference. For example; there have been two cohort studies conducted in tertiary facilities in China. In these studies, 24 of 27 cases and 5 of 9 cases with monogenic IBD had IL10R signaling defects. The percentage of colitis with IL10R signaling defects in Asia is higher than in Europe (35–41).

**Table 1B:** Cohort studies reporting the prevalence of monogenic IBD in pediatric IBD patients.

**References**	**Ratio %**	**Age**	**Number**	**First approach of sequencing technology**	**Objects**
Crowley et al. (35) (Canada)	3.1	<18 yr	31/1,005	WES; all cases	• All pediatric IBD patients diagnosed and followed at a single center (2003–2015) • Only IBD primary care and referral center in the Greater Toronto Area, Canada
	13.8	<2 yr	4/29		
	7.7	<6 yr	11/142		
	2.3	6< age <18	20/863		
Uchida et al. (36) (Japan)	13.9	<18 yr	15/108	TGPS; all cases	• Early onset diarrhea refractory to conventional therapy • IBD; 95 cases, refractory diarrhea; 13 cases • 15 institutions in Japan
	17.1	<2 yr	7/41		
	9.9	<6 yr	8/81		
	25.9	6< age <18	7/27		
Lega et al. (37) (2019) (Italy)	12.9	<18 yr	12/93	WES; *n* = 16 TGPS; *n* = 69 Sanger sequence; *n* = 9	• VEO-IBD and patients with early onset IBD with severe/atypical phenotypes • 2 main pediatric GI center (2008–2017) and 9 external gastroenterology facilities in Italy
	12.7	<2 yr	7/55		
	11.5	<6 yr	10/87		
	33.3	6< age <18	2/6		
Charbit-Henrion et al. (38) (Europe)	31.9	<18 yr	66/207	WES; *n* = 51 TGPS; *n* = 167 Sanger sequence; *n* = 32	• Chronic diarrhea developed before the age of 6 with severe disease course requiring immunosuppressive treatments, surgery, and/or parenteral nutrition and additional patients with disease onset after the age of 6 years in the presence of a FH suggestive Mendelian inheritance or of a disease course refractory to treatment • 45 institutions in Europe
	41	<2 yr	59/144		
	33.5	<6 yr	62/185		
	18.2	6< age <18	4/22		
Fang et al. (39) (China)	25.8	<2 yr	8/31	WES; *n* = 6 TGPS; *n* = 12	• All VEOIBD cases diagnosed in a single center (2005–2017)
	16.7	<6 yr	9/54		• A tertiary center in China
Kammermeier et al. (40) (UK)	30.6	<2 yr	19/62	WES; *n* = 37 TGPS; *n* = 17 Sanger sequence; *n* = 8	• All IOIBD cases managed at a single center over the past 20 years • A tertiary center in UK
Ye et al. (41) (China)	71.1	<2 yr	27/38	WES; *n* = 17 Sanger sequence; *n* = 21	• All IOIBD cases diagnosed based on Porto criteria at a single (2015–2017) • A tertiary center in China

Recently, Crowley et al. reported data from a cohort of over 1,000 children with IBD at a single center in Canada (35). This center has the distinction of being the only IBD primary care and referral center in this area, and most children with IBD in this region are diagnosed and followed up at this center. All children with IBD from mild to severe underwent WES, and pathogenic variants of monogenic IBD were found to occur in 7.8% of children younger than 6 years of age and 13.8% of children younger than 2 years of age. This cohort includes a variety of racial groups, which may be representative of the real world. Based on these previous cohorts, monogenic IBD has been found to be about 1.5 times as prevalent in children younger than 2 years of age with IBD as in children younger than 6 years of age with IBD. The prevalence of monogenic IBD is at least 10–15% in children younger than 2 years of age with IBD and 7%-10% in children younger than 6 years of age with IBD, however, regional differences should be considered.

### IBD Onset at the Age 6 and Older

The majority of the studies on monogenic IBD have focused on children younger than 6 years of age. Recently, there have been several reports dealing with children older than 6 years of age. The aforementioned large cohort from Canada showed that 2.5% of pediatric IBD occurring between the ages of 6 and 18 years was monogenic IBD (35). Three other cohort studies have reported that the incidence of monogenic IBD in severe refractory IBD cases in individuals aged between 6 and 18 years was ~20–30%. Recent reports suggest that physicians should be aware of the presence of rare variants causing monogenic IBD in children diagnosed older than 6 years of age. IL10/R, TTC7A and IKBKG are known to cause severe colitis in early infancy (12, 42, 43), whereas XIAP deficiency, IBD with Hermansky–Pudlak Syndrome, and IBD caused by mutation in TNFAIP3 can occur in a wide range of ages from infancy to adulthood (32, 44–46). CGD-colitis with an autosomal recessive pattern of inheritance occurs more frequently in younger individuals (47). However, CYBC1 deficiency was recently identified occurring in CGD-colitis in adolescents aged on average 12.2 years (range 7-19 years) (48).

## Identifying Monogenic IBD in Clinical Practice

The key indicators of monogenic IBD in clinical practice are age of onset, family history, and extraintestinal manifestations based on individual gene defects. Infant IBD (under 2 years of age at onset) and VEOIBD are more likely to be monogenic IBD, and aggressive genetic testing is recommended. Concerning family history, there are two issues to be aware of. The first is “family relationships” including consanguinity. The second is “family diseases” as it is not uncommon for disease presentation to differ among family members despite having the same gene defect (49). Therefore, it is important to ask for a detailed family history of autoimmune diseases of other organs and infectious diseases besides IBD.

In the following sections, we discuss comorbidities. Extraintestinal manifestation is a critical indicator of monogenic IBD. Physical findings and comorbidities of which physicians should be aware at the initial physical examination and during follow-up are shown in Figure 1C. Recurrent and atypical infections are commonly found in monogenic IBD, since a large proportion of underlying monogenic disorders are primary immune deficiencies. Specific skin lesions, lymphadenopathy, and hepatosplenomegaly on physical examination are also physical findings that should be raise suspicions of monogenic IBD. There are some monogenic disorders with characteristic complications that are relatively easy to differentiate. Severe perianal disease, folliculitis, and/or arthritis in early infancy suggest the presence of IL-10 signaling defects disorders (50). In cases of extraintestinal manifestation with autoimmune anemia, type 1 diabetes mellitus, autoimmune thrombocytopenia, autoimmune thyroiditis, interstitial pneumonia, or other multi-organ autoimmune disorders, IBD may be one of the symptoms of IPEX syndrome caused by FOXP3 deficiency or IPEX-like syndrome caused by mutations in CTLA4, LRBA, STAT1, STAT3, or CARMIL2 (14, 51–56). GSD-1b and Niemann pick C are monogenic metabolic disorders that could cause IBD (16, 57). In many cases, findings such as hypoglycemic symptoms and hepatomegaly point to underlying metabolic diseases in early infancy, and IBD occurs after a diagnosis of metabolic disorders.

## XIAP Deficiency and Chronic Granulomatous Disease

We sometimes have difficulty diagnosing monogenic IBD because of the lack of severe complications. The most frequent disorders in this category are XIAP deficiency and CGD (47, 58, 59). These disorders should be considered in children younger than 6 years of age with IBD, and should be excluded using functional analysis described below (Table 1A) or targeted sequencing for patients older than 6 years of age with IBD refractory to conventional therapy or requiring surgical intestinal resection.

Regarding XIAP deficiency, flow cytometry to evaluate XIAP expression is valuable for diagnostic screening tests. However, there are patients with XIAP deficiency who may have a normal protein expression but lack function. Therefore, we need assays for evaluating the TNF production of monocytes stimulated by L18-MDP (muramyl dipeptides) (60). For the diagnosis of CGD, dihydrorhodamine 1,2,3 (DHR-123) is useful. Besides, neutrophil/monocyte respiratory burst tests should be considered, because patients with CGD caused by NCF4 with normal or mildly impaired DHR-123 (33).

Early diagnosis is beneficial for patients with both XIAP deficiency and CGD. XIAP can develop life-threatening hemophagocytic lymphohistiocytosis (HLH), therefore HSCT should be considered early before HLH occurs (8, 61). CGD patients have a high risk of infection, therefore anti-tumor necrosis factor (TNF) alpha is contraindicated as it may increase this risk (62).

## Conclusions and Future Directions

In this paper, we have reviewed the essential features of monogenic IBD, as seen in clinical practice. The use of WES and TGPS has led to improved diagnosis of severe refractory IBD as monogenic IBD. In the future, more genetic mutations will be discovered, and the role of immunity in intestinal inflammation will be elucidated using techniques including transcriptomics, proteomics, and metabolomics. It is further expected that appropriate treatments for these monogenic disorders will be developed. Because monogenic IBD is very rare, the collaboration of specialized facilities around the world is required for the further development of individualized treatment strategies. The interNational Early Onset Pediatric IBD Cohort Study (NEOPICS) (http://www.neopics.org/) and (http://www.VEOIBD.org) keep working to identify the causes and to develop new treatments together with international pediatric gastroenterologists and scientists from academic centers around the world.

## Author Contributions

RN conceived, designed, and drafted manuscript. AM drafted, edited, and revised manuscript. RN and AM approved final version of manuscript. All authors contributed to the article and approved the submitted version.

## Conflict of Interest

The authors declare that the research was conducted in the absence of any commercial or financial relationships that could be construed as a potential conflict of interest.

## Abbreviations

CD: Crohn disease
CGD: Chronic granulomatous diseases
FGDS: Familial GUCY2C diarrhea syndrome
GSD-1b: Glycogen storage disease type 1b
GWAS: Genome-wide association studies
HSCT: Hematopoietic stem cell transplantation
IBD: Inflammatory bowel disease
IBDU: Inflammatory bowel disease-unclassified
IPEX, Immune dysregulation, polyendocrinopathy, enteropathy: X-linked
LRBA: Lipopolysaccharide-responsive and beige-like anchor
NEMO: Nuclear factor-kappa B essential modulator
TGPS: Targeted genome panel sequencing
TNF: Tumor necrosis factor
UC: Ulcerative colitis
VEOIBD: Very early onset inflammatory bowel disease
WES: Whole exome sequences
XIAP: X-linked inhibitor of apoptosis protein.
